# Root Exudate-Mediated Interactions Among Maize, Sorghum, and Soybean During Germination and Early Development

**DOI:** 10.17912/micropub.biology.001632

**Published:** 2025-07-14

**Authors:** Shiran Ben Zeev, Risha K. Yaragarla, Marin M. Cooney, Deerdra J. Stauch, Guojie Wang

**Affiliations:** 1 Department of Plant Science, Pennsylvania State University, State College, Pennsylvania, United States

## Abstract

Designing sustainable cropping systems requires incorporating ecological principles, particularly those that improve system-level function through species diversity. Intercropping, growing two or more species in close proximity, offers a promising strategy to enhance productivity and reduce input reliance. We tested whether root exudates from maize, sorghum, and soybean influence conspecific and heterospecific germination and seedling growth. Using hydroponically collected exudates, we found species-specific effects: maize exudates reduced germination across species, soybean showed selective inhibition with broad growth promotion, and sorghum was largely unresponsive. These early exudate-mediated interactions may shape compatibility among intercrop species and inform the design of ecologically grounded cropping systems.

**Figure 1. Response of Maize, Soy, and Sorghum seeds and seedlings to conspecific and heterospecific root exudates:**
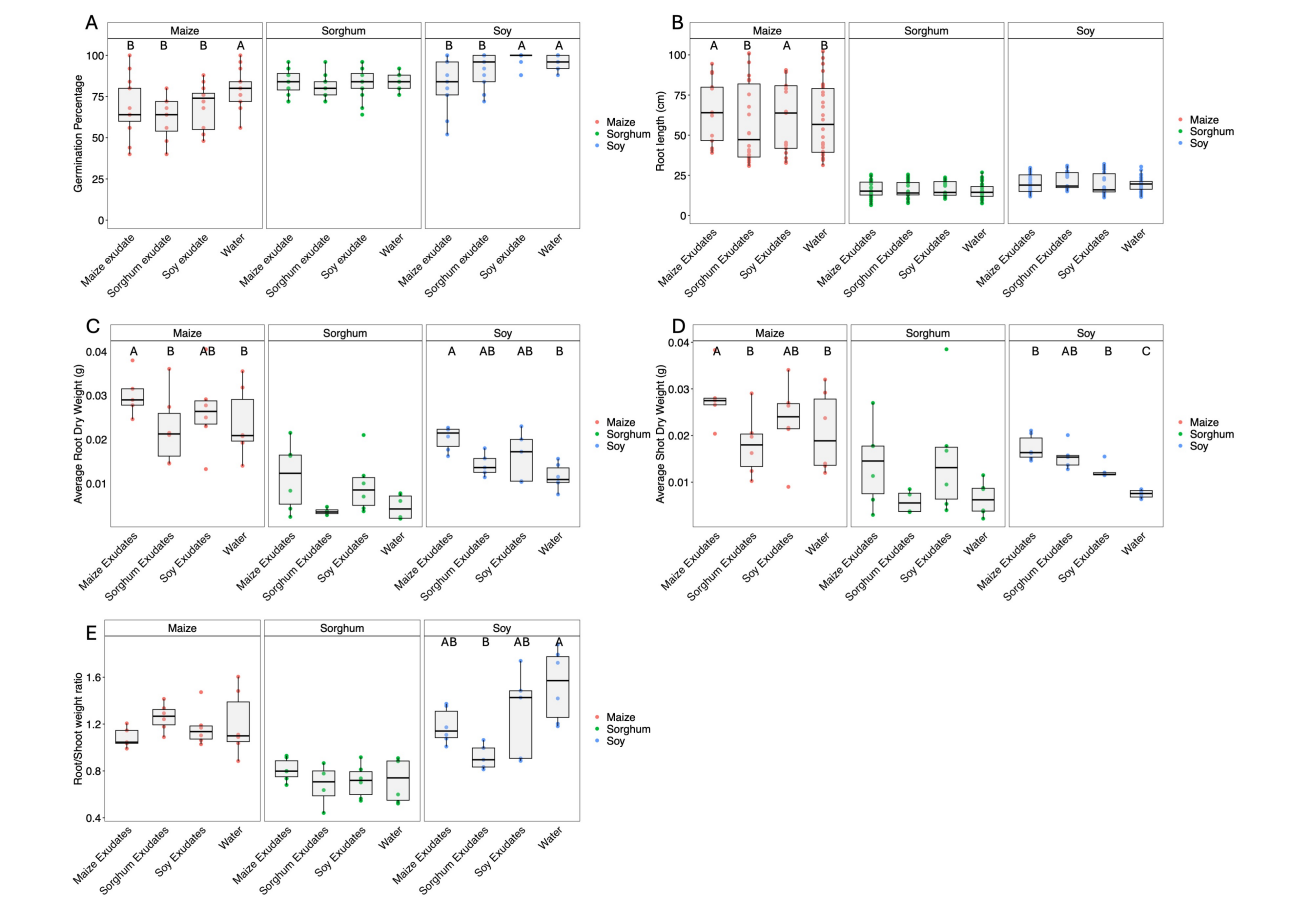
A. Final germination percentage, B. Total root length (per plant), C. Root Dry weight, D. Shoot Dry weight, E. Root/Shoot weight ratio of corn, sorghum, and soy seedlings grown in root exudates of 14-day-old corn, sorghum, and soybean plants and water. Different letters indicate significant differences (P<0.05) in a Tukey-Kramer HSD test.

## Description

Designing sustainable cropping systems requires incorporating ecological principles, particularly those that improve system-level function through species diversity (Altieri et al., 2015). Most annual crops are grown in monoculture, which maximizes input efficiency but often compromises long-term resilience and soil health (Bourke et al., 2021; Power & Follett, 1987). Intercropping - growing two or more species in close proximity - offers a promising alternative. It can enhance nutrient acquisition, alter root architecture, and improve resource partitioning, potentially increasing productivity and reducing reliance on external inputs (Brooker et al., 2015; Li et al., 2013).

A key but underexplored mechanism in intercrop performance is root exudation. Roots release a diverse set of organic compounds—including sugars, amino acids, phenolics, and phytohormones—that influence nutrient cycling, microbial communities, and neighboring plants (Scavo et al., 2019; Vives-Peris et al., 2020). While exudates are known to mediate plant–plant interactions in natural systems, their roles in early crop establishment and interspecific signaling remain poorly understood. These chemically mediated effects may influence seed germination, seedling vigor, and early competitive dynamics.

Here, we tested whether root exudates from three functionally distinct crops: maize ( *Zea mays* L.), sorghum ( *Sorghum bicolor* (L.) Moench), and soybean ( *Glycine max* (L.) Merr.)—alter conspecific and heterospecific seed performance. These species are common in U.S. warm-season cropping systems and differ in photosynthetic pathway (C3-soybean vs. C4-maize and sorghum), nutrient strategy (nitrogen-fixing vs. non-fixing), and root architecture (taproot vs. fibrous) (Cheriere et al., 2023; Li et al., 2006). To isolate chemical effects from microbial and physical effects, we collected root exudates in a hydroponic system and applied them to seeds and seedlings under controlled conditions (Kawasaki et al., 2018; Wheeldon et al., 2022). We tested two hypotheses: (1) germination rates would differ among exudate treatments, and (2) seedling growth would respond in a species-specific manner to exudate origin.

**Seed Germination responses varied among species and exudate types.** A two-way ANOVA revealed significant effects of exudate treatment (F₍3,159₎ = 5.82, P = 0.00085), species (F₍2,159₎ = 66.66, P < 0.0001), and their interaction (F₍6,159₎ = 3.07, P = 0.0071) on germination percentage. For maize, germination was significantly reduced in all exudate treatments compared to water, including in conspecific exudates (mean = 69.4% ± 2.7 SE vs. 79.3% ± 2.7 SE in water), supporting a potential autotoxic effect as a mechanism for density regulation. Soybean germination was also reduced in heterospecific (maize and sorghum) exudates, but not in conspecific exudates, indicating selective inhibition. In contrast, sorghum germination remained statistically unchanged across treatments (P > 0.9), potentially reflecting greater tolerance or detoxification capacity.

**Seedling development exhibited species-specific growth promotion.** Two-way ANOVAs revealed significant effects of exudate treatment on both root (F₍3,53₎ = 4.57, P = 0.0064) and shoot biomass (F₍3,53₎ = 4.58, P = 0.0064), as well as strong species effects in both cases (Root: F₍2,53₎ = 54.49, P < 0.0001. Shoot, F₍2,53₎ = 19.88, P = 3.62e–07). No significant species × treatment interaction was detected for either trait. In maize, root biomass was significantly greater in maize exudates (mean = 0.029 g ± 0.0008 SE) compared to sorghum exudates (0.0145 g ± 0.0009 SE; P = 0.0508) and water (0.0204 g ± 0.0006 SE, P = 0.0209). Shoot biomass followed a similar trend, though pairwise contrasts were not significant (means ranged from 0.0275–0.0383 g). Soybean seedlings exhibited the greatest overall biomass accumulation, with shoot biomass significantly higher in maize and soy exudates (up to 0.0387 g) compared to water (0.0276 g). In contrast, sorghum root and shoot biomass showed no significant treatment effects (P = 0.08), though numerical trends paralleled those of maize, with slightly enhanced growth in maize and soybean exudates.

**Biomass allocation was relatively stable across treatments.** With no significant interaction for root: shoot ratio (P = 0.99). Maize and sorghum maintained consistent allocation patterns regardless of exudate type. However, soybean exhibited a significantly lower root: shoot ratio in sorghum exudates compared to water (mean = 1.20 vs. 1.41; P < 0.05), suggesting a shift toward shoot growth when exposed to heterospecific chemical signals.

Together, these results demonstrate that root exudates can mediate both inhibitory and facilitative interactions during early plant development, and that responses are species-specific. The consistent reduction in maize germination across all exudates, including conspecifics, supports a hypothesis of autotoxicity, which may regulate intraspecific competition and spacing. Soybean’s selective germination inhibition, combined with strong growth responses to all exudates, suggests an early filtering phase followed by plastic growth promotion, possibly reflecting differential sensitivity across developmental stages. Sorghum’s limited responsiveness at both germination and seedling stages could reflect a more conservative or delayed response strategy.

These exudate-mediated interactions are likely ecologically relevant in intercropping systems. Soybean’s positive growth response to maize exudates may promote facilitation in cereal x legume combinations, while maize’s potential autotoxicity could hinder tight spacing or replanting strategies. Our findings suggest that root exudates contribute to early-stage compatibility among species and should be considered when designing intercrop pairings for sustainable production.

## Methods

Plant Material and Exudate Collection- We used three crop species common to U.S. intercropping systems: maize ( *Zea mays* , line C49647883192, Brevant USA), sorghum ( *Sorghum bicolor* , line SSA181 BMR 6, Seedway USA), and soybean ( *Glycine max* , line AE4950, Revere Seeds USA). Seeds were germinated in hydroponic culture and grown in a greenhouse at Penn State University under controlled conditions (28°C/18°C Day/Night, 12 h photoperiod, LED lighting). Seedlings were grown in 1 mL/L Miracle-Gro solution for 11 days, after which they were transferred to tap water (at the four-leaf stage) for exudate collection. Six plants per species were grown per cycle, and the three largest seedlings were selected to ensure consistent exudate contributions.

Plants were incubated in tap water for 48 hours in sterilized containers, after which the solution was filtered (0.22 µm) and stored at 4°C. Fresh exudates were used for each experimental block to minimize degradation.

**Germination Assay -** To test germination responses, seeds of all three species were placed on moistened filter paper in sterile 100 mm Petri dishes containing 15 mL of either root exudate (from maize, sorghum, or soybean) or deionized water (control). Plates were sealed with parafilm, wrapped in foil, and incubated at 28°C under a 12 h light/dark cycle. Germinated seeds (radicle emergence >1 mm) were counted and removed daily for 7 days. The experiment followed a randomized complete block design with four blocks of 48 plates each (12 plates per treatment per species), ensuring all species were tested in all exudate types in each block.

**Seedling Growth Assay** - We used the paper roll method to assess early seedling development. Seeds were placed between two sheets of germination paper soaked in either root exudate or water, rolled vertically, and incubated for 7 days at 28°C. Each roll contained four seeds of the same species and treatment. The experiment was repeated in three independent blocks, with eight rolls per treatment per block (24 rolls per treatment in total). At harvest, we measured total root length, counted root tips, and separated shoots and roots for biomass measurements. Samples were dried at 60°C for 72 hours and weighed with a precision was 0.1 mg. The 28°C incubation temperature is commonly used for germination and early growth in all three species and approximates optimal physiological conditions for warm-season crops.

**Statistical Analysis- Statistical Analysis** — All analyses were conducted in R (v4.4.1). Germination data were analyzed using generalized linear models (binomial family), and seedling growth data were analyzed using linear models (ANOVA) with species, exudate treatment, and their interaction as fixed effects, and block as a factor. Post hoc comparisons were performed using Tukey's HSD tests via the emmeans package, and significance groupings were extracted using the multcomp package. Residuals were evaluated for normality. All analyses were performed using the following R packages: dplyr, lme4, emmeans, multcomp, and tidyr. Data visualization was conducted using ggplot2.
